# Carriage prevalence of *Neisseria meningitidis* in China, 2005–2022: a systematic review and meta-analysis

**DOI:** 10.1186/s12879-022-07586-x

**Published:** 2022-07-07

**Authors:** Mengmeng Yue, Juan Xu, Jianxing Yu, Zhujun Shao

**Affiliations:** 1 School of Public Health, Nanjing Medical University, Nanjing, China; 2 Department of Respiratory Infectious Diseases, National Institute for Communicable Disease Control and Prevention, Chinese Center for Disease Control and Prevention, No. 155# Changbai Road, Chang Ping, Beijing, People’s Republic of China

**Keywords:** *Neisseria meningitidis*, Prevalence, China, Meta-analysis

## Abstract

**Introduction:**

*Neisseria meningitidis* (*Nm*) is a major cause of meningitis and septicemia. Most people are infected with latent infections or are carriers. We aimed to estimate the carriage prevalence of *Nm* in China.

**Methods:**

We did a systematic review of published work to assess the prevalence of meningococcal carriage in China. The quality assessment was conducted by the risk of bias tool according to Damian Hoy’s study. We estimated pooled proportions of carriage and its 95% confidence interval (95% CI) using fixed effect model for studies with low heterogeneity and random effect model for studies with moderate or high heterogeneity. Subgroup analyses were also conducted by region and age group.

**Results:**

In total, 115 studies were included. The quality evaluation grades of all included documents were medium or high grade. The weighted proportion of carriage was 2.86% (95% CI: 2.25–3.47%, *I*^*2*^: 97.7%, *p* = 0). The carriage prevalence of *Nm* varied between provinces, ranged from 0.00% (95% CI: 0.00–0.66%) to 15.50% (95% CI: 14.01–16.99%). Persons aged 15 years and older had the highest carriage 4.38% (95% CI: 3.15–5.62%, *I2*: 95.4%, *p* < 0.0001), and children under 6 years of age had the lowest carriage 1.01% (95% CI: 0.59–1.43%, *I2*: 74.4%, *p* < 0.0001). In positive carriers, serogroup B (41.62%, 95% CI: 35.25–48.00%, *I*^*2*^: 98.6%, *p* = 0) took up the highest proportion, and serogroup X (0.02%, 95% CI: 0.00–0.09%, *I*^*2*^: 0.00%, *p* = 1) accounted for the lowest proportion.

**Conclusion:**

The meningococcal carriage in China was estimated low and varied by region and age group. Understanding the epidemiology and transmission dynamics of meningococcal infection in insidious spreaders is essential for optimizing the meningococcal immunization strategies of the country.

**Supplementary Information:**

The online version contains supplementary material available at 10.1186/s12879-022-07586-x.

## Introduction

*Neisseria meningitidis* (*Nm*), a gram-negative bacterium that colonizes 10% of the human nasopharynx and spreads through the respiratory droplets of infected people, can cause invasive meningococcal disease (IMD), such as meningitis and septicemia [1, 2]. According to the structure and characteristics of capsular polysaccharides, *Nm* strains are divided into 12 serogroups (A, B, C, W, X, Y, Z, E, H, I, K, L) and non-groupable serogroups [3]. It is generally believed that six groups (*Nm* A, B, C, W, Y and X) are the main causes of IMD and that non-groupable *Nm* is not pathogenic.

Globally, the incidence and mortality of meningococcal disease have continued to decrease since 1990, although differences in age and geographic distribution remained [4, 5]. In 2020, the incidence was 0.56 per 100,000 population in Spain and 0.17 per 100,000 population in Brazil [5]. During 2015–2019, the incidence rate of meningococcal disease in China was 0.078 per million persons, and the case fatality rate was 11.82% [6]. The reported cases of meningitis in China are mainly people aged 10–19 years, accounting for 34.15% (111/325) of the total reported cases of meningococcal disease, followed by people aged 1–9 years, accounting for 29.54% (96/325) [7].

Invasive cases are relatively rare in meningococcal infected cases while most cases are asymptomatic [8]. The phenomenon of asymptomatic colonization in the upper respiratory tract mucosa is known as carriage [2]. The colonization of *Nm* in the nasopharynx is the initial step in IMD development [9]. The meningococcus of the patient is usually obtained through close contact with carriers rather than patients [10]. Estimates of carriage prevalence are important for studying the dynamics of carriage and disease and for understanding the potential effect of control programs, such as vaccination, on the transmission of meningococci.

In the African meningitis belt, the carriage prevalence of *Nm* ranged from 0.595% in infants to 1.94% at age 10 [11]. In European countries, the highest carriage prevalence was 23.7% in 19-year old [8]. In the Americas, the prevalence among adolescents and young adults, especially university students and males, was higher than that of other populations [12]. These indicate that differences exist between regions and age groups. The overall carriage prevalence of *Nm* between 2000 and 2013 in China was 2.7% (95%CI: 2.0–3.5%), but the regional distribution and age distribution was unclear [13]. Understanding the distribution of meningococcal carriage in regions and age groups is critical to understanding the spread of *Neisseria meningitidis*.

Knowing the carriage prevalence can understand the dynamics of the spread of bacteria in the population, which is the important evidence for evaluating, planning, and implementing intervention measures, such as vaccine immunization. Currently, the meningococcal vaccines marketed in China include Group A meningococcal polysaccharide vaccine (MPV-A), Group A and group C meningococcal polysaccharide vaccine (MPV-AC), Group A, C, Y, and W135 meningococcal polysaccharide vaccine (MPV-ACWY), Group A and group C meningococcal polysaccharide conjugate vaccine (MPCV-AC), Group A and group C meningococcal polysaccharide conjugate and Haemophilus type b conjugate combined vaccine (MPCV-AC-Hib) [14, 15]. There is evidence that meningococcal polysaccharide conjugate vaccines (MPCVs) can reduce nasopharyngeal meningococcal bacteria carriage and have the ability to induce herd protection [16, 17]. There is no group B meningococcal vaccine in China. American CDC’s Advisory Committee on Immunization Practices (ACIP) recommends to vaccinate the quadrivalent meningococcal conjugate vaccine (MenACWY) for teenagers aged 11 or 12 years, and to boost immunization at the age of 16 [18]. Knowing the carriage prevalence of *Nm* can indirectly indicate the IMD prevalence. Meanwhile, understanding the difference in the carriage prevalence of different age groups can help to adjust the immunization strategy.

We conducted this systematic review and meta-analysis to evaluate the meningococcal carriage prevalence in China and to learn the distribution of *Nm* and serogroup proportions in positive carriers. Learning the regions and age groups with high level of carriage is important for understanding the transmission dynamics and determination of target population for vaccination. It is of significance for the development of new vaccines, such us serogroup B vaccines, to find out the serogroup with the highest proportion in positive carriers.

## Methods

### Search strategy and data sources

This review was conducted in accordance with the PRISMA 2020 statement [19] (Additional file 1: Table S1) to identify articles reporting the carriage of *Nm* in different provinces in China published between 1st January 2005 and 30th April 2022. We searched five databases [China national knowledge infrastructure (CNKI), Wanfang Data Knowledge Service Platform (Wanfang), China Science and Technology Journal Database (VIP), China Biology Medicine disc (CBMdisc) and PubMed] using the following medical subject headings (MeSH) and text words: “Cerebrospinal meningitis”, “Meningococcal meningitis”, “Meningococcal Infections”, “Meningitis”, “*Neisseria meningitidis*”, “Neisseria”, “Meningococcal”, and “carriage”.

### Inclusion and exclusion criteria

Studies were considered for inclusion if they met the following criteria: (1) the studies reported pharyngeal carriage of all meningococcal serogroups from different provinces in China; (2) the subjects of these studies must be healthy populations; (3) the studies were peer-reviewed and published between 1st January 2005 (when MPV-AC was included in the national immunization program) and 30th April 2022; (4) the studies were published in English or Chinese.

Studies were ineligible for inclusion if they met the following items: (1) case reports, case–control reports, outbreak investigations, reviews and other meta-analyses; (2) Studies that reported carriage among cases or close contacts of cases; (3) Studies that only reported the carriage prevalence of a single serogroup of meningococci; (4) studies with incomplete data; (5). Studies that reported the evaluation of the effect of antibiotics or post-chemical prophylaxis research results; (6) Duplicate studies including the same samples.

### Data extraction and classification

Study selection (including screening titles and abstracts and assessment through full text review) and data collection were independently conducted by two authors (YMM and XJ). If disagreement occurred, we sought for the recommendation of the third researcher (SZJ). The data extracted from eligible studies included the following aspects: title, first author, publication year, region, research time, sampling methods, lab methods, the number of age groups, the number of carriers and sample size. Provinces were classified into seven geographical regions [20], i.e. northeast (Heilongjiang, Jilin and Liaoning), north (Beijing, Hebei, Inner Mongolia and Shanxi), east (Anhui, Fujian, Jiangsu, Jiangxi and Shandong), south (Guangdong, Guangxi and Hainan), central (Henan, Hubei and Hunan), northwest (Gansu, Ningxia, Qinghai, Shaanxi and Xinjiang), and southwest (Guizhou, Sichuan and Yunnan). Due to the different methods of age groupings reported in different literatures, the median age of each age group in the literature was used for the age grouping of subgroup analysis. The reported age groups of study participants were divided into three groups, i.e. 0–6 years, 7–14 years, and ≥ 15 years, since children aged 0–6 years are required to be vaccinated in the National Immunization Schedule.

### Quality assessment

The quality assessment of the included studies was independently conducted by two reviewers (YMM and XJ). The risk of bias tool was used to assess the quality of selected studies according to Damian Hoy’s study, including external validity (Items 1 to 4) and internal validity (Items 6 to 10) [21]. Items included the sampling frame of the sample, the sampling methods, the nonresponse bias, the case definition, the data collected, lab method, and data source. Each question answered “yes” received one point, while the “no” answer for each question received zero. In addition, each question answered “unknown” got 0.5 points. The risk of bias was classified as high (0–5 score), medium (5.5–8 score) and low (8.5–10 score).

### Statistical analyses

All statistical analyses were performed in R software (version 4.1.2, Auckland University, USA). We used the metaprop function in the meta package to pool proportions of included studies. Subgroup analyses were conducted by province, region and age group. The Higgins *I*^*2*^ test was used to measure heterogeneity between studies. Heterogeneity was classified as low (0 < *I*^*2*^ ≤ 50%), moderate (50% < *I*^*2*^ ≤ 75%) and high (75% < *I*^*2*^ ≤ 100%). A fixed effect model was performed for studies with low heterogeneity, while a random effect model was used for studies with moderate or high heterogeneity. Funnel plots and Egger’s test were used to evaluate possible publication bias. If publication bias exists, the trim-and-fill method was performed to evaluate the impact of publication bias on the results. Sensitivity analysis was performed to assess the stability of the results by calculating the combined carriages and 95% CIs after excluding each selected study.

## Results

### Study screening

**Fig. 1:**
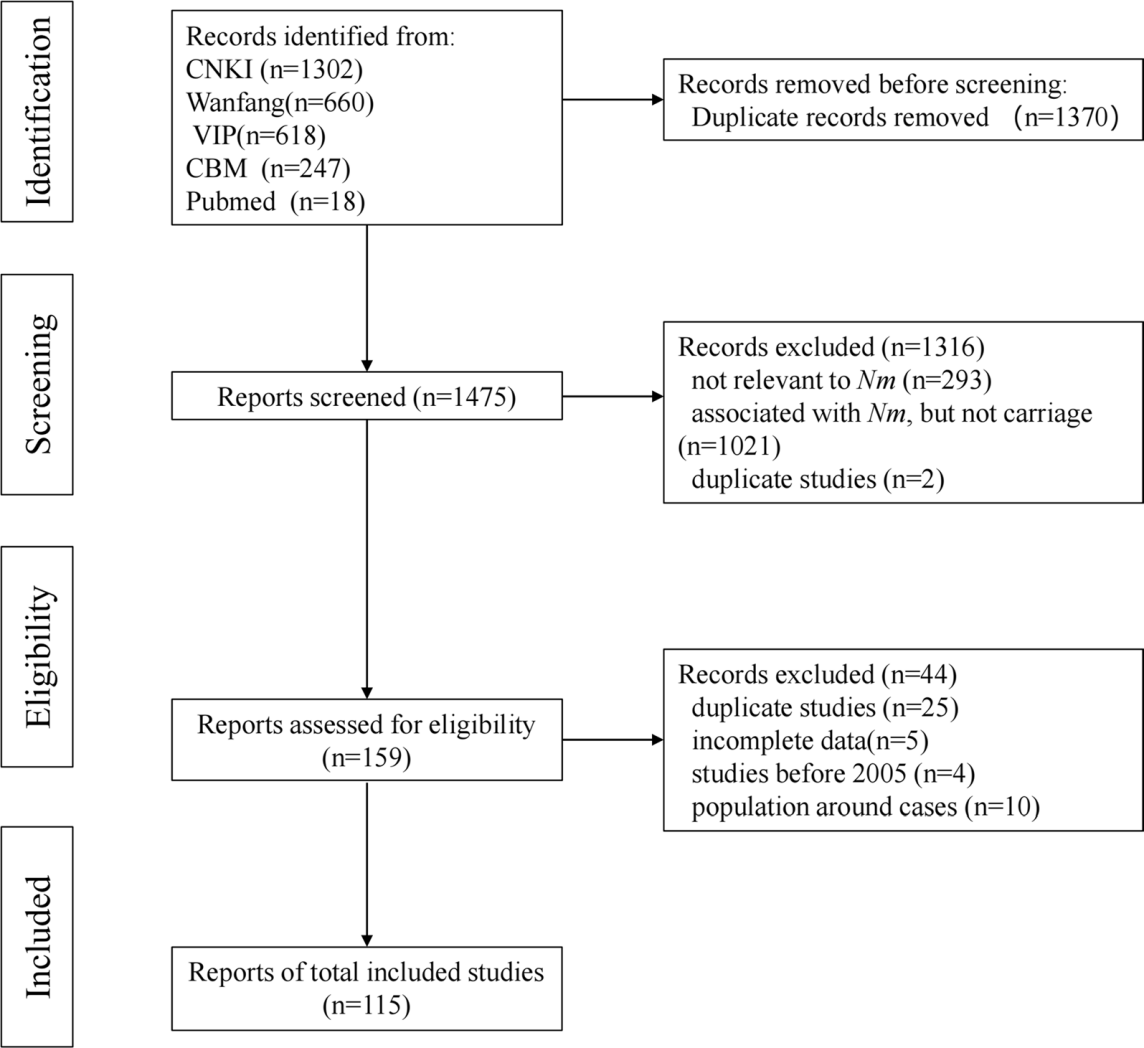
Flow chart of study selection

Overall, 2845 records were identified from five databases based on the search strategy. After removing 1370 duplicated records, 1475 studies remained. 1316 records were excluded after screening the titles and abstracts, i.e., 293 records not relevant to *Nm*, 2 duplicated studies, and 1021 studies associated with *Nm* but not carriage. In the full text screening process, 159 studies were screened, and 44 studies excluded, i.e., 25 duplicated studies, 4 studies published before 2005, and 15 studies carriage data not reportable. Overall, 115 studies reporting the carriage of *Nm* in different provinces of China were included in the systematic review and meta-analysis (Fig. 1).

### Characterization and quality assessments of included studies

Among the 115 included studies, 114 studies reporting the carriage prevalence of *Nm* of 28 provinces in China were included regional subgroup analysis (Table 1). 66 studies were cross sectional, 48 were serial cross sectional, and one study was a combination of cross sectional and longitudinal. 57 studies reported on the carrying status of meningococci in different age groups using different grouping methods. 55 studies reported the carriage prevalence with different sampling methods: cluster sampling, cluster stratified random sampling, random sampling, multistage stratified random Sampling, simple random sampling, stratified cluster sampling and stratified random sampling. 104 studies used the isolation and culture of meningococcus as the identification standard, and 10 of them also used PCR as the identification standard.

**Table 1:** Characterization of included 115 studies

ID	Study	First author	Province	Publication time	Study time	Sampling method	Testing method	NO. of age groups	Cases	Sample size	Carriage prevalence
1	Yueyun Lan-2012 [22]	Yueyun Lan	Zhejiang	2012	1985–2010	UN	UN	UN	1762	7649	23.04%
2	Ruichun Ding-2013 [23]	Ruichun Ding	Hunan	2013	2011–2012	UN	Isolation and culture	7	1	442	0.23%
3	Xiumin Liang-2018 [24]	Xiumin Liang	Yunnan	2018	2014	Cluster sampling	Isolation and culture	4	16	240	6.67%
4	Hongfei Zhang-2010 [25]	Hongfei Zhang	Inner Mongolia	2010	2007	Stratified random sampling	Isolation and culture	UN	13	221	5.88%
5	Weijun Hu-2020 [26]	Weijun Hu	Shaanxi	2020	2016–2017	Cluster sampling	Isolation and culture, PCR	UN	110	1539	7.15%
6	Yueqi Wang-2017 [27]	Yueqi Wang	Shaanxi	2017	2016	Cluster sampling	Isolation and culture, PCR	6	64	998	6.42%
7	Tingting Yang-2019 [28]	Tingting Yang	Shanxi	2019	2016–2017	UN	Isolation and culture	6	25	649	3.9%
8	Honglian Lai-2011 [29]	Honglian Lai	Fujian	2011	2010	Stratified random sampling	Isolation and culture	UN	0	335	0%
9	Jialing Zhang-2019 [30]	Jialing Zhang	Jiangsu	2019	2014–2016	Random sampling	Isolation and culture	8	150	1265	11.86%
10	Xianping He-2013 [31]	Xianping He	Sichuan	2013	2010	UN	Isolation and culture	UN	2	273	0.73%
11	Zunyu Liu-2016 [32]	Zunyu Liu	Shandong	2016	2008–2015	Random sampling	Isolation and culture	7	24	2362	1.02%
12	Yingtong Wang-2015 [33]	Yingtong Wang	Hebei	2015	2006–2013	Stratified cluster sampling	Isolation and culture	7	293	28,447	1.03%
13	Zhenwu Liu-2017 [34]	Zhenwu Liu	Anhui	2017	2015	Stratified cluster sampling	Isolation and culture	6	23	1093	2.10%
14	Haiying Deng-2010 [35]	Haiying Deng	Hainan	2010	2006	Stratified random sampling	Isolation and culture	UN	9	744	1.21%
15	Min Cui-2013 [36]	Min Cui	Guangdong	2013	2009–2011	Stratified random sampling	Isolation and culture	UN	6	791	0.8%
16	Xinghua Wu-2010 [37]	Xinghua Wu	Guangxi	2010	2008	Stratified cluster sampling	Isolation and culture	UN	22	367	5.99%
17	Deshan Qiu-2016 [38]	Deshan Qiu	Shandong	2016	2013–2014	Stratified random sampling	Isolation and culture	7	8	996	0.80%
18	Weiping Jiang-2014 [39]	Weiping Jiang	Jiangsu	2014	2011–2012	UN	Isolation and culture	UN	17	703	2.42%
19	Hongna Chu-2016 [40]	Hongna Chu	Hebei	2016	2013–2014	Stratified random sampling	Isolation and culture	7	16	442	3.62%
20	Yihong Zhou-2012 [41]	Yihong Zhou	Jiangsu	2012	2011	UN	Isolation and culture	8	14	320	4.38%
21	Bin Jia-2016 [42]	Bin Jia	Beijing	2016	2009–2013	UN	Isolation and culture	9	34	1224	2.78%
22	Hengcai Niu-2018 [43]	Hengcai Niu	Beijing	2018	2016	Cluster stratified random sampling	Isolation and culture	9	11	252	4.37%
23	Fei He-2020 [44]	Fei He	Zhejiang	2020	2013–2017	Volunteer recruiting	Isolation and culture, PCR	7	329	2807	11.72%
24	Weihua Xue-2016 [45]	Weihua Xue	Hebei	2016	2015–2015	Stratified random sampling	Isolation and culture	7	11	255	4.31%
25	Qingxiu Zheng-2019 [46]	Qingxiu Zheng	Beijing	2019	2015–2017	Cluster sampling	Isolation and culture	9	14	783	1.79%
26	Lei Geng-2017 [47]	Lei Geng	Hebei	2017	2015–2016	Stratified random sampling	Isolation and culture	7	11	215	5.12%
27	Yihong Liao-2017 [48]	Yihong Liao	Fujian	2017	2014–2016	UN	Isolation and culture	UN	0	600	0%
28	Fangqin Xie-2016 [49]	Fangqin Xie	Fujian	2016	2011	UN	Isolation and culture	UN	0	263	0%
29	Yunfeng Hu-2011 [50]	Yunfeng Hu	Fujian	2011	2009	Stratified random sampling	Isolation and culture	UN	0	188	0%
30	Shiguo Liang-2011 [51]	Shiguo Liang	Heilongjiang	2011	2009	Random sampling	Isolation and culture	UN	11	210	5.24%
31	Yongfei Yan-2018 [52]	Yongfei Yan	Hebei	2018	2009–2015	Stratified random sampling	Isolation and culture	7	100	3528	2.83%
32	Xiaolei Tang-2010 [53]	Xiaolei Tang	Qinghai	2010	2007	UN	Isolation and culture	5	11	480	2.30%
33	Maolin Wang-2011 [54]	Maolin Wang	Shandong	2011	2007–2010	Simple random sampling	Isolation and culture	7	14	1470	0.95%
34	Zhijun Wang-2010 [55]	Zhijun Wang	Henan	2010	2004–2008	UN	Isolation and culture	5	32	855	3.74%
35	Yemin Qi-2014 [56]	Yemin Qi	Hebei	2014	2000–2013	UN	Isolation and culture	UN	232	4865	4.77%
36	Lin Luan-2014 [57]	Lin Luan	Jiangsu	2014	2005–2012	Cluster sampling	Isolation and culture, PCR	UN	26	4043	0.64%
37	Caixia Hao-2010 [58]	Caixia Hao	Sichuan	2010	2008–2009	UN	Isolation and culture	7	6	212	2.83%
38	Jingzhi Gao-2019 [59]	Jingzhi Gao	Hubei	2019	2008–2018	UN	UN	UN	93	2818	3.30%
39	Rongwei Lan-2014 [60]	Rongwei Lan	Guangxi	2014	2011	Cluster stratified random sampling	Isolation and culture	5	112	1311	8.54%
40	Yafei Wang-2013 [61]	Yafei Wang	Shandong	2013	2012	UN	UN	UN	5	430	1.16%
41	Qian Liu-2013 [62]	Qian Liu	Henan	2013	2010–2012	UN	Isolation and culture	UN	99	1653	5.99%
42	Xufang Ye-2017 [63]	Xufang Ye	Guizhou	2017	2006	Stratified random sampling	Isolation and culture	UN	3	726	0.41%
43	Ling Yuan-2012 [64]	Ling Yuan	Fujian	2012	2009	UN	Isolation and culture	UN	3	727	0.4%
44	Huanzhang Yuan-2012 [65]	Huanzhang Yuan	Guangdong	2012	2008–2010	UN	Isolation and culture	7	7	737	0.95%
45	Dan Xiao-2011 [66]	Dan Xiao	Liaoning	2011	2002–2009	UN	UN	UN	8	1990	0.4%
46	Fengyun Cheng-2012 [67]	Fengyun Cheng	Anhui	2012	2009	Stratified random sampling	Isolation and culture	UN	2	80	2.5%
47	Xiang Sun-2018 [68]	Xiang Sun	Jiangsu	2018	2014–2015	UN	Isolation and culture	UN	76	755	10.07%
48	Haitao Liu-2016 [69]	Haitao Liu	Beijing	2016	2013–2015	Stratified random sampling	Isolation and culture	UN	41	756	5.42%
49	Suxin Xu-2013 [70]	Suxin Xu	Hebei	2013	2012	Stratified random sampling	Isolation and culture	7	16	420	3.81%
50	Junrong Lu-2013 [71]	Junrong Lu	Hebei	2013	2012	Stratified random sampling	Isolation and culture	5	8	382	2.09%
51	Junhong Li-2010	Junhong Li	China	2010	2009	UN	UN	UN	92	9743	0.94%
52	Manshi Li-2010 [72]	Manshi Li	Shandong	2010	2008–2009	UN	Isolation and culture	7	13	1097	1.19%
53	Xuan Deng-2018 [73]	Xuan Deng	Zhejiang	2018	2006–2017	UN	UN	UN	4	2524	0.16%
54	Lijun Chen-2012 [74]	Lijun Chen	Guangdong	2012	2006–2008	UN	Isolation and culture	UN	1	705	0.14%
55	Xiaoping Yan-2010 [75]	Xiaoping Yan	Sichuan	2010	2006–2008	Random sampling	Isolation and culture	6	13	540	2.4%
56	Heng Yuan-2010 [76]	Heng Yuan	Sichuan	2010	2005–2008	UN	Isolation and culture	8	61	4369	1.40%
57	Jingjing Wu-2020 [77]	Jingjing Wu	Shandong	2020	2008–2018	UN	Isolation and culture	UN	34	3827	0.89%
58	Yan Wang-2016 [78]	Yan Wang	Liaoning	2016	2004–2013	UN	UN	UN	41	5197	0.79%
59	Shenxia Chen-2013 [79]	Shenxia Chen	Zhejiang	2013	2011–2013	Stratified random sampling	Isolation and culture	UN	5	152	3.29%
60	Quwen Li-2014 [80]	Quwen Li	Fujian	2014	2012	UN	Isolation and culture	UN	2	806	0.25%
61	Xiaofeng Yang-2007 [81]	Xiaofeng Yang	Hunan	2007	2006	Cluster sampling	Isolation and culture	7	10	367	2.72%
62	Xiaoqing Fu-2006 [82]	Xiaoqing Fu	Yunnan	2006	2005	UN	Isolation and culture	UN	14	979	1.43%
63	Taiping Yang-2007 [83]	Taiping Yang	Guangdong	2007	2006	Cluster sampling	Isolation and culture	5	9	352	2.56%
64	Xiaochun Li-2007 [84]	Xiaochun Li	Sichuan	2007	2005	UN	Isolation and culture	6	10	336	2.98%
65	Yushan Fan-2008 [85]	Yushan Fan	Hebei	2008	2001–2007	UN	Isolation and culture	UN	60	1792	3.35%
66	Fang Guo-2007 [86]	Fang Guo	Zhejiang	2007	2001–2006	UN	Isolation and culture	7	92	1779	5.17%
67	Sujie Shi-2006 [87]	Sujie Shi	Jiangsu	2006	2005	Random sampling	UN	9	8	470	1.70%
68	Shuxian Zhang-2009 [88]	Shuxian Zhang	Liaoning	2009	2006–2008	UN	Isolation and culture	UN	43	618	6.96%
69	Ye Chen-2007 [89]	Ye Chen	Liaoning	2007	2005	UN	UN	UN	13	229	5.68%
70	Qunwen Wen-2006 [90]	Qunwen Wen	Guangdong	2006	2005	Cluster sampling	Isolation and culture	7	10	717	1.39%
71	Changyan Ju-2008 [91]	Changyan Ju	Guangdong	2008	2005–2006	UN	Isolation and culture	7	55	1255	4.4%
72	Guohua Li-2006 [92]	Guohua Li	Shanxi	2006	2005	Random sampling	Isolation and culture	9	11	940	1.17%
73	Jianmin Zhang -2009 [93]	Jianmin Zhang	Zhejiang	2009	2003–2008	UN	Isolation and culture	3	87	1507	5.77%
74	Youju Jia-2008 [94]	Youju Jia	Qinghai	2008	2006–2007	UN	Isolation and culture	UN	11	450	2.44%
75	Yan Yang-2008 [95]	Yan Yang	Sichuan	2008	2007	UN	Isolation and culture, PCR	2	13	230	5.65%
76	Chunyuan Cao-2009 [96]	Chunyuan Cao	Fujian	2009	2006–2007	UN	Isolation and culture	UN	1	251	0.40%
77	Meng Yang-2007 [97]	Meng Yang	Jiangxi	2007	2005	UN	Isolation and culture	9	29	1441	2.01%
78	Xiaoqing Liu-2009 [98]	Xiaoqing Liu	Jiangxi	2009	2005–2007	UN	Isolation and culture	UN	55	3312	1.66%
79	Zhenglong Zhong-2008 [99]	Zhenglong Zhong	Jiangsu	2008	2005–2007	Random sampling	Isolation and culture	7	10	193	5.18%
80	Wen Lu-2008 [100]	Wen Lu	Heilongjiang	2008	2005	Random sampling	Isolation and culture	UN	3	230	1.30%
81	Jing Lv-2006 [101]	Jing Lv	Hubei	2006	2000–2005	UN	Isolation and culture	UN	213	4921	4.33%
82	Zhenyu Qian-2009 [102]	Zhenyu Qian	Hebei	2009	2007–2008	Stratified random sampling	Isolation and culture	7	163	7460	2.18%
83	Xiaoping Wang-2007 [103]	Xiaoping Wang	Anhui	2007	2005–2006	Simple random sampling	Isolation and culture	5	82	1868	4.39%
84	Xiujuan Yan-2006 [104]	Xiujuan Yan	Hainan	2006	2005	Random sampling	Isolation and culture	UN	14	617	2.27%
85	Tie Song-2007 [105]	Tie Song	Guangdong	2007	2005	UN	UN	UN	17	2413	0.70%
86	Lianfei Zhao-2007 [106]	Lianfei Zhao	Ningxia	2007	2007	Random sampling	UN	UN	0	210	0%
87	Jianying Yang-2005 [107]	Jianying Yang	Gansu	2005	2005	UN	Isolation and culture	9	2	742	0.27%
88	Ping Lin-2007 [108]	Ping Lin	Fujian	2007	2005	Random sampling	Isolation and culture	UN	1	190	0.530%
89	Huake Yang-2006 [109]	Huake Yang	Guangdong	2006	2005	UN	Isolation and culture	UN	0	226	0%
90	Shuhua Luo-2006 [110]	Shuhua Luo	Guangdong	2006	2005	UN	Isolation and culture	7	1	616	0.16%
91	Yan Teng-2009 [111]	Yan Teng	Jilin	2009	2008	Cluster sampling	Isolation and culture	7	0	210	0%
92	Yonggeng Zou-2009 [112]	Yonggeng Zou	Hunan	2009	2008	UN	Isolation and culture	7	1	240	0.42%
93	Huanying Gu-2009 [113]	Huanying Gu	Hebei	2009	2008	Stratified random sampling	Isolation and culture	UN	4	348	1.15%
94	Xinghua Wu-2009 [114]	Xinghua Wu	Guangxi	2009	2008	UN	Isolation and culture	UN	32	864	3.7%
95	Weijun Wang-2008 [115]	Weijun Wang	Chongqing	2008	2007	UN	Isolation and culture	UN	3	638	0.47%
96	Yinqi Sun-2008 [116]	Yinqi Sun	Hebei	2008	2007	UN	Isolation and culture	7	58	3618	1.60%
97	Rongna Huang-2009 [117]	Rongna Huang	Sichuan	2009	2007	Random sampling	Isolation and culture, PCR	5	11	999	1.1%
98	Qingmei Cong-2009 [118]	Qingmei Cong	Shandong	2009	2007–2008	UN	Isolation and culture	7	6	840	0.71%
99	Jianwen Yin-2007 [119]	Jianwen Yin	Yunnan	2007	2006	Cluster sampling	Isolation and culture	6	21	1249	1.68%
100	Lihua Ren-2008 [120]	Lihua Ren	Inner Mongolia	2008	2006	Stratified random sampling	Isolation and culture	7	8	210	3.81%
101	Yun Gong-2009 [121]	Yun Gong	Fujian	2009	2006	Stratified random sampling	Isolation and culture	UN	4	652	0.6%
102	Jun Wang-2007 [122]	Jun Wang	Ningxia	2007	2006	UN	Isolation and culture	UN	4	214	1.9%
103	Zuokui Xiao-2009 [123]	Zuokui Xiao	Shandong	2009	2007–2008	UN	Isolation and culture	UN	73	4836	1.52%
104	Xinchang Luo-2006 [124]	Xinchang Luo	Fujian	2006	2005	UN	Isolation and culture	UN	2	360	0.55%
105	Xin Li-2007 [125]	Xin Li	Inner Mongolia	2007	2005	UN	Isolation and culture	UN	27	711	3.80%
106	Hai Wang-2007 [126]	Hai Wang	Anhui	2007	2005	Random sampling	Isolation and culture	4	32	1047	3.06%
107	Lv You-2006 [127]	Lv You	Guizhou	2006	2005	UN	isolation and culture	9	17	904	1.88%
108	Meizhen Liu-2007 [128]	Meizhen Liu	Guangdong	2007	2005	UN	Isolation and culture	UN	7	1077	0.65%
109	Yan Zhang-2021 [129]	Yan Zhang	Shandong	2021	2009–2020	Recruitment	Isolation and culture, PCR	6	136	16,848	0.81%
110	Jinjun Luo-2021 [130]	Jinjun Luo	Hubei	2021	2013–2018	UN	Isolation and culture	7	370	4477	8.26%
111	Na Xie-2021 [131]	Na Xie	Xinjiang	2021	2012–2019	Cluster stratified random sampling	Isolation and culture, PCR	UN	351	2264	15.5%
112	Xiaohong Zhou-2021 [132]	Xiaohong Zhou	Jiangsu	2021	2018	UN	Isolation and culture	UN	7	411	1.70%
113	Man Jiang-2021 [133]	Man Jiang	Jiangsu	2021	2017–2018	Multistage stratified random sampling	Isolation and culture	8	15	772	1.94%
114	Chen Chen-2021 [134]	Chen Chen	Yunnan	2021	2020	Random Sampling	Isolation and culture, PCR	UN	17	1076	1.58%
115	Yunyi Zhang-2022 [135]	Yunyi Zhang	Zhejiang	2022	2015–2020	UN	Isolation and culture, PCR	UN	17	2827	0.64%

Most (85.22%, 98/115) included studies received a medium score of the quality assessment (Additional file 1: Fig. S1). No study received a high risk-of bias score. The target population of 115 studies was not well representative of the national population. Most (97.39%, 112/115) studies did not cover a sufficient period of time (≥ 1 year) to account for seasonal variation and 58.26% (67/115) of the included studies did not report whether they used random sampling.

### Carriage prevalence by region

**Fig. 2:**
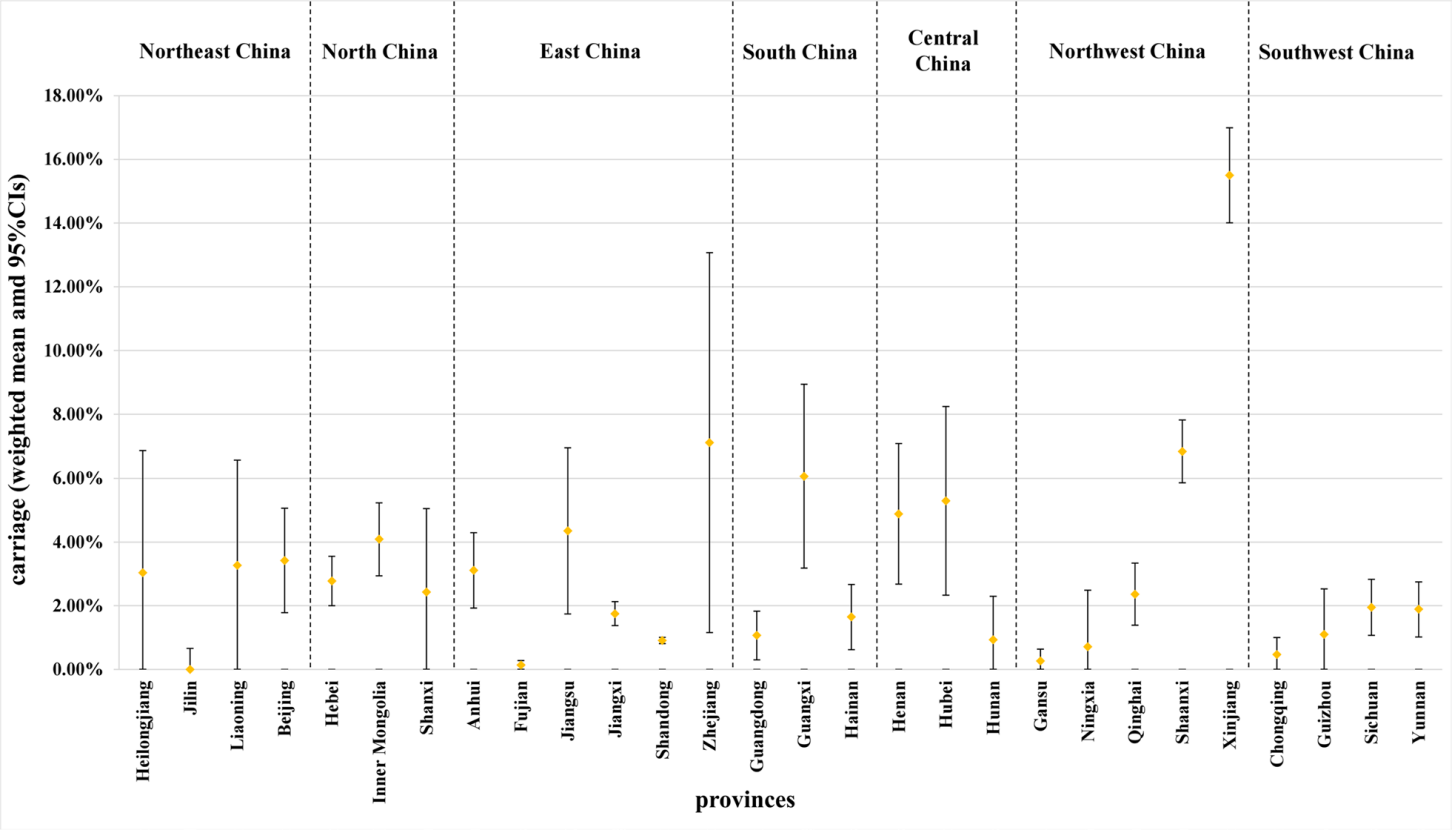
Carriage prevalence by province. The weighted mean was calculated with random effect model. Yellow diamonds represent the weighted mean, and solid black lines represent the 95% CIs

The overall carriage prevalence of *Nm* of all 115 studies was 2.86% (95% CI: 2.25–3.47%, *I*^*2*^: 97.7%, *p* = 0) with random effect model. In the results of subgroup analysis by province (Fig. 2), the meningococcal carriage rate ranged from 0.00% (95% CI: 0.00–0.66%) in Jilin in northeast China to 15.50% (95% CI: 14.01–16.99%) in Xinjiang in northwest China. In the results of subgroup analysis by region (Table 2), the meningococcal carriage prevalence ranged from 1.65% (95% CI: 1.10–2.20%) in Southwest China to 4.48% (95% CI: 0.91–8.05%) in Northwest China.

**Table 2:** Carriage prevalence by region

Item	Southwest China	Northeast China	South China	East China	Central China	Northwest China	North China
Studies	14	7	15	41	8	8	21
Total number of cases	207	119	302	3168	819	553	1156
Sample size	12,771	8684	12,792	74,096	15,773	6897	57,518
I^2^ (P value)	76.3% (*p* < 0.01)	90.8% (*p* < 0.01)	93.7% (*p* < 0.01)	98.7% (*p* = 0)	98.3% (*p* < 0.01)	98.6% (*p* < 0.01)	93.8% (*p* < 0.01)
Model	Random	Random	Random	Random	Random	Random	Random
Weighted carriage	1.65%	2.66%	2.12%	2.82%	3.62%	4.48%	3.00%
95%CI	1.10–2.20%	0.52–4.80%	0.92–3.31%	1.48–4.16%	1.74–5.50%	0.91–8.05%	2.39–3.62%

### Carriage prevalence by age

The age group data were divided into 3 age groups according to the different age groupings of each study (Table 3). Random effect mode was used to generate the weighted carriage rate of each age group. As shown in Table 2, the highest carriage was 4.38% (95% CI: 3.15–5.62%) in age group ≥ 15 years old, and the lowest carriage was 1.01% (95% CI: 0.59–1.43%) in 0–6 years age group.

**Table 3:** Carriage prevalence by age

Item	0–6 years	7–14 years	≥ 15 years
Studies	56	57	56
Total number of cases	296	667	1662
Sample size	26,491	37,763	39,633
I^2^ (P value)	74.4%(*p* < 0.0001)	82.2% (*p* < 0.0001)	95.4% (*p* < 0.0001)
Model	Random	Random	Random
Weighted carriage	1.01%	1.81%	4.38%
95%CI	0.59–1.43%	1.32–2.30%	3.15–5.62%

### The proportion of *N. meningitidis* serogroups in positive cases

As shown in Table 4, random effect model was used to calculated the proportion of *Nm* serogroups, except NmX and NmY, in positive cases of carriage studies. The proportion of meningococcal serogroup in positive cases ranges from 0.02% (0.00–0.09%) of serogroup X to 41.62% (35.25–48.00%) of serogroup B.

**Table 4:** The proportion of *N. meningitidis* serogroups in positive cases

Item	A	B	C	W	X	Y	Others and non-groupable
Studies	95	95	95	95	95	95	95
Total number of cases	486	3124	508	195	43	116	1791
Sample size	6263	6263	6263	6263	6263	6263	6263
I^2^ (P value)	86.4% (*p* < 0.0001)	98.6% (*p* = 0)	84.1% (*p* < 0.0001)	57.4% (*p* < 0.0001)	0.0% (*p* = 1)	29.8% (*p* = 0.0044)	98.1% (*p* = 0)
Model	Random	Random	Random	Random	Fixed	Fixed	Random
Weighted carriage	9.70%	41.62%	11.22%	2.13%	0.02%	0.03%	21.77%
95%CI	6.93–12.47%	35.25–48.00%	8.51–13.93%	1.16–3.09%	0.00–0.09%	0.00–0.10%	16.62–26.93%

### Publication bias and sensitivity analysis

**Fig. 3:**
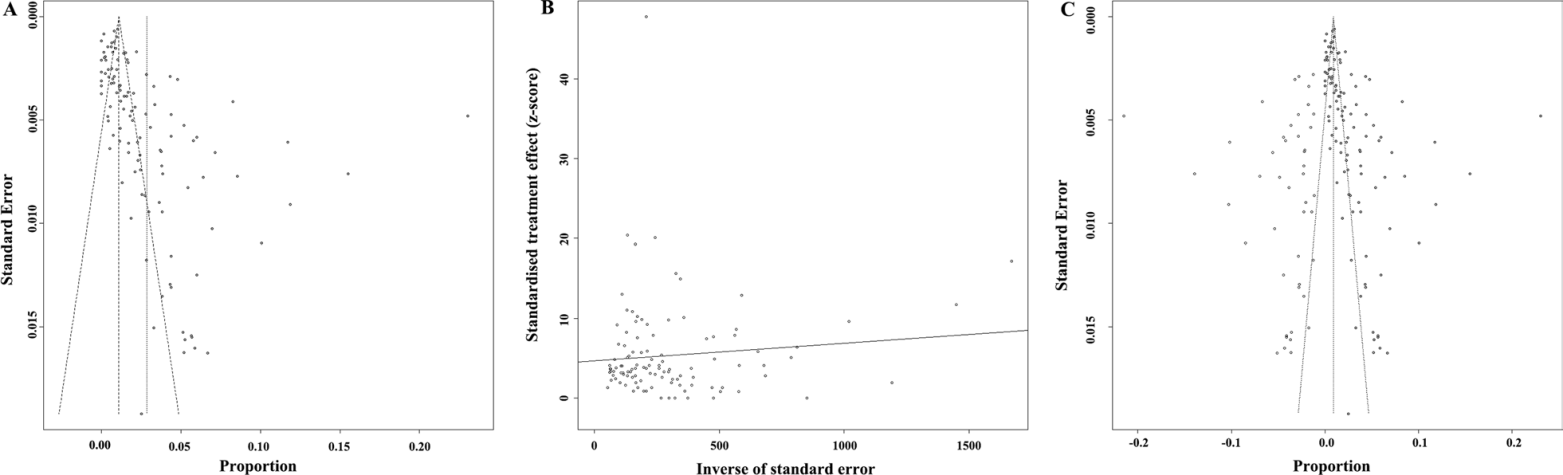
Analysis of publication bias of the 124 included studies. Panel **A** Funnel plot; Panel **B** Egger’s test; Panel **C** Trim and Fill. Hallow circles show data points of added studies and filled circles show data points of included studies

We used funnel plots and Egger’s linear regression to assess the publication bias of all included studies. The result of the funnel plot, which was asymmetric (Fig. 3A), and the P value of Egger’s test (Fig. 3B, P < 0.0001) illustrated the presence of publication bias. The weighted mean carriage rate was 0.91% (95%CI: 0.18–1.64%, Q = 9937.12, *p* = 0, *I*^*2*^ = 98.4%, 95% CI of *I*^*2*^: 98.3–98.5%) after adding 45 studies by the trim-and-fill method (Fig. 3C). The results of the sensitivity analysis (Additional file 1: Fig. S2) illustrated that the combined carriages and 95% CIs after excluding each selected study did not show much change. The results of the meta-analysis were stable and steady.

## Discussion

At present, this is the first systematic review and meta-analysis to describe the regional distribution and age distribution of meningococcal carriage prevalence in healthy people in China. We estimated the overall carriage rate to be 2.85% (95% CI: 2.24–46%), which is lower than that reported in Cuba (31.9%), America (24%) and Brazil (21.5%) [12]. Limited nasopharyngeal swab sampling collection and insufficient laboratory testing capacity in different regions may contribute to the low carriage prevalence of *Nm*. The transportation of samples may also affect the carriage prevalence of *Nm*.

More than half of the studies were retrieved from East and North China, with the largest number of studies from Fujian Province of East China. A part (8.06%) of the research subjects were from rural areas [71, 83, 95, 102, 106, 113, 116, 119, 122, 136]. The majority of the study subjects included people of all ages, and only 2 were conducted on primary and middle school students [93, 95]. During 2006 and 2014, the provinces with the most cases of meningitis in China included Anhui (cases = 159) and Jiangsu (cases = 70) provinces in East China and Hebei Province (cases = 61) in North China [137]. Between 2015 and 2019, there were still many cases of meningitis reported in Hebei in North China while cases in Southwest and Northeast were fewer than that of other regions [6]. In a study analyzing the results of surveillance of meningococcal disease in China in 2009, 9743 subjects in eight provinces or cities were tested, and the carriage rate was 0.94% (92/9743), in which Hebei in North China was the province with the highest carriage rate [138].

According to the results of the age subgroup analysis, the meningococcal carriage rates of age group 7–14 and the age group ≥ 15 years old were higher than those of children (0–6 years). In the African meningitis belt, the carriage prevalence of individuals aged 5–19 years were significantly higher than that of other age groups [11]. Since 2010, the meningococcal serogroup A conjugate vaccine (MenAfriVac) has been introduced in 26 countries of the African meningitis belt for individuals aged 1–29 years [139]. In European countries, the carriage prevalence increased from 4.5% in infants to a peak of 23.7% in 19-year-old adolescents and then decreased in adulthood to 7.8% in adults aged 50 [8]. This demonstrates the success of the immunization program of meningococcal serogroup C conjugate (MCC) for children under 18 in UK [140]. In China, the basic immunization population of the five meningococcal vaccines that have been marketed are children aged 0–6 [141]. It is important to improve vaccine strategies to determine whether it is necessary to booster immunization with meningococcal meningitis vaccines among people aged ≥ 7 years.

In our study, the highest and lowest proportion of *N. meningitidis* serogroups in positive meningococcal carriers was NmB with 41.62% (35.25–48.00%) and NmX with 0.02% (0.00–0.09%). Globally, serogroup B was the foremost cause of invasive meningococcal disease in America, Europe, and the western Pacific [142, 143]. At present, vaccines marked in China includes NmA, NmC, NmW and NmY vaccines except NmB vaccines [14, 15]. It is urgent for the development of serogroup B vaccines.

The results of the funnel plot and trim-and-fill method indicate that there is publication bias in this study. As 114 studies were regional and small-scale studies, the target population of these studies was not well representative of the national population (Additional file 1: Fig. S1). This review includes only published studies without unpublished literature whose results may be not significant.

A limitation of this review is that there is no unified standard on sample collection and laboratory testing methods, which can cause bias that impacts the results of meta-analysis. Inconsistent diagnostic methods and a lack of diagnostic kits may lead to underestimation or misinformation of the data reported in the study.

Understanding the carriage prevalence of *Nm* in generalizable populations contributes to providing evidence for further improvement of meningococcal vaccine and vaccination strategies. This is important for the prevention of meningitis and development of vaccines in China in the future.

## Conclusion

In summary, the meningococcal carriage in China was estimated low and varied by region and age group. Based on our findings, we suggest that the surveillance on epidemic cerebrospinal meningitis among generalizable populations in each province and region in China should be enhanced. The age distribution of meningococcal carriage highlights the importance of monitoring and booster immunization among teenagers aged ≥ 7 years.

## Supplementary Information

**Additional file 1: Table S1.** PRISMA checklist. **Fig S1.** Quality assessment of the included studies. **Fig S2.** Forest plot of sensitivity analysis.

## Data Availability

The datasets generated and analyzed during the current study are available from the corresponding author on reasonable request.

## Abbreviations

*Nm*: *Neisseria meningitidis*
IMD: Invasive meningococcal disease
CNKI: China national knowledge infrastructure
Wanfang: Wanfang Data Knowledge Service Platform
VIP: China Science and Technology Journal Database
CBMdisc: China Biology Medicine disc
MeSH: Medical subject headings
MPV-A: Group A meningococcal polysaccharide vaccine
MPV-AC: Group A and group C meningococcal polysaccharide vaccine
MPV-ACWY: Group A, C, Y, and W135 meningococcal polysaccharide vaccine
MPCV-AC: Group A and group C meningococcal polysaccharide conjugate vaccine
MPCV-AC-Hib: Group A and group C meningococcal polysaccharide conjugate and Haemophilus type b conjugate combined vaccine
